# Circulating Neutrophil Dysfunction in HBV-Related Acute-on-Chronic Liver Failure

**DOI:** 10.3389/fimmu.2021.620365

**Published:** 2021-02-25

**Authors:** Wei Wu, Shanshan Sun, Yijie Wang, Ruihong Zhao, Haotang Ren, Zhiwei Li, Hong Zhao, Yi Zhang, Jifang Sheng, Zhi Chen, Yu Shi

**Affiliations:** ^1^ State Key Laboratory for Diagnosis and Treatment of Infectious Diseases, National Clinical Research Center for Infectious Diseases, Collaborative Innovation Center for Diagnosis and Treatment of Infectious Diseases, The First Affiliated Hospital, Zhejiang University School of Medicine, Hangzhou, China; ^2^ Division of Hepatobiliary and Pancreatic Surgery, Department of Surgery, First Affiliated Hospital, School of Medicine, Zhejiang University Cytometry, Hangzhou, China; ^3^ Department of Laboratory Medicine, The First Affiliated Hospital, Zhejiang University School of Medicine, Hangzhou, China; ^4^ Key Laboratory of Clinical In Vitro Diagnostic Techniques of Zhejiang Province, Hangzhou, China

**Keywords:** hepatitis B virus, acute-on-chronic liver failure, neutrophil, phagocytosis, NET

## Abstract

**Background and Aims:**

Acute-on-chronic liver failure (ACLF) is characterized by systemic inflammation accompanied by defective anti-bacterial immunity. The role of neutrophils in immune derangement of ACLF has not been fully elucidated. This study is aimed to characterize the role of circulating neutrophils in HBV-related ACLF patients.

**Methods:**

Quantitative, phenotypic, transcriptomic, and functional alterations of circulating neutrophils were compared in ACLF and non-ACLF subjects and analyzed for associations with short-term outcomes. Interventional experiments were performed to test the impact on ACLF-patient neutrophil function *in vitro*.

**Results:**

Circulating absolute neutrophil count was significantly increased in patients with ACLF and was an independent risk factor for 28-day mortality. ACLF-patient neutrophils differentially expressed a panel of surface markers (include TLR-1, TLR-2, TLR-4, CEACAM-1 and FPR1), as well as a distinct transcriptomic signature. ACLF-neutrophils displayed significantly impaired phagocytosis but an increased capacity to form neutrophil extracellular traps (NETs), which was more pronounced in patients with poor outcome. Healthy neutrophils mimicked functional characteristics of ACLF counterpart after co-cultured with plasma from ACLF patients. The oxidative burst and cytokine production capacities remained unchanged. Plasma GM-CSF, IL-6, IL-8, IL-10, and IP-10 levels, as well as lipopolysaccharide (LPS) concentration, were markedly elevated in ACLF patients but not DAMP molecules HMGB-1 and HSP70. Finally, a glycolysis inhibitor, 2-deoxy-glucose, reduced NET formation of ACLF patients’ neutrophils.

**Conclusions:**

Circulating ACLF-patient neutrophils exhibit alterations in number, phenotype, gene expression and function, which was associated with poor outcome and shaped by the ACLF circulatory environment. Inhibiting glycolysis can reverse neutrophil dysfunction in ACLF patients.

## Introduction

Chronic hepatitis B virus (HBV) infection remains a major global health burden, with 350 million chronically infected individuals worldwide (1, 2). In addition to hepatocellular carcinoma (HCC) and decompensated cirrhosis, acute-on-chronic liver failure (ACLF) is another cause of death in patients with chronic HBV infection (3). Prior studies have shown that abrupt and intensive systemic inflammation (SI) with associated immune paresis plays a key role in the pathogenesis of ACLF (4).

Neutrophils are a key component of the circulating innate immune cell population and serve as critical effector cells among the first line of defense against bacterial infections or tissue damage (5). Neutrophils are recruited to sites of inflammation by chemokines and mitochondria-derived formyl peptides, where they eliminate pathogens or remove cell debris and participate in tissue healing (5). There is increasing evidence suggesting that neutrophils play a critical role in hepatic inflammation. It has been demonstrated that both the number and ratio of circulating neutrophils are sensitive prognostic markers of the severity and outcomes of patients with cirrhosis or liver failure (6, 7). Neutrophil dysfunction, including impaired phagocytic activity, altered spontaneous oxidative burst, reduced complement expression, and decreased intracellular killing, is also observed in cirrhosis (8, 9), alcoholic hepatitis (10) and acute liver failure (11). Such functional defects strongly affect mortality (10, 12, 13). However, studies of neutrophils in ACLF are still scarce.

We previously demonstrated that the neutrophil-to-lymphocyte ratio (NLR) predicts disease progression and mortality in HBV-ACLF patients, consistent with other studies (14, 15). In this study, the phenotypic, transcriptomic, and functional alterations were characterized in circulating neutrophils from HBV-ACLF patients. And we further tested whether glycolysis is involved in functional changes of ACLF-neutrophils and its inhibition leads to reverse of functional aberrance.

## Subjects and Methods

### Subjects

To confirm the absolute neutrophil count (ANC) as an independent risk factor for mortality, we first included a retrospective cohort of 667 subjects, which incorporating 63 patients with hepatitis B virus related acute-on-chronic liver failure (HBV-ACLF), 34 healthy controls (HC), 95 patients with compensated liver cirrhosis (CLC), and 475 patients with decompensated liver cirrhosis (DLC) admitted to the First Affiliated Hospital of Zhejiang University from May 2014 to August 2015 (cohort A). For the subsequent experiments, we prospectively enrolled a separate cohort of study subjects, including 67 patients with hepatitis B virus related acute-on-chronic liver failure (HBV-ACLF), 21 healthy controls (HC) and 28 patients with compensated liver cirrhosis (CLC) from the First Affiliated Hospital of Zhejiang University between August 2018 and July 2020 (cohort B). Information of 28-day and 3-month prognosis was obtained from medical records, telephone contact or a visit. The detailed individual information is shown in **Table 1** and **Supplementary Table 1**. The diagnosis of HBV-ACLF was established by the criteria proposed by the Chinese Cohort on the Study of Severe Hepatitis B (COSSH) (16). HE was defined and graded by West Haven criteria (17). MELD scores were calculated according to the Malinchoc formula (18).

**Table 1:** Baseline characteristics of study population in Cohort A.

Variables	HC (N=34)	CLC (N=95)	DLC (N=475)	ACLF (N=63)	P value(HC Vs ACLF)	P value (CLC Vs ACLF)	P value (DLC Vs ACLF)
**Age (years)**	48 (18)	58(17)	54(14)	52(20)	0.395	0.010	0.512
**Male No. (%)**	19 (56)	78(82)	372(78)	57(91)	0.000	0.145	0.024
**HBV DNA [Lg_10_ (copies/ml)]**	–	0(3.9)	0(4.5)	3.8(5.0)	0.000	0.001	0.002
**HBeAg positivity No. (%)**	–	27(28)	136(29)	20(31.7)	0.000	0.718	0.584
**ALT (IU/L)**	16(11)	25(21)	29(25)	97(165)	0.000	0.000	0.000
**AST (IU/L)**	19(6)	32(23)	40(37)	124(159)	0.000	0.000	0.000
**Albumin (g/L)**	45.8 ± 4.1	38.6 ± 4.3	31.2 ± 6.4	31.1 ± 4.9	0.000	0.000	0.852
**Serum bilirubin (µmol/L)**	11 (6)	13 (10)	24 (25)	329 (147)	0.000	0.000	0.000
**INR**	0.9(0.1)	1.0(0.1)	1.2(0.3)	1.9(1.0)	0.000	0.000	0.000
**Creatinine (μmol/L)**	68 (27)	69(16)	70(25)	69(32)	0.231	0.714	0.694
**Serum sodium (mmol/L)**	142(3)	142(3)	141(3)	137(7)	0.000	0.000	0.000
**Platelet count (10^9^/L)**	193(73)	108(83)	64(62)	73(73)	0.000	0.001	0.366
**MELD score**	–	6.4(0.7)	7.2(1.5)	10.5(5.4)	0.000	0.000	0.000
**Acute jaundice No. (%)**	–	0(0)	50(11)	63(100)	0.000	0.000	0.000
**Ascites No. (%)**	–	0(0)	316(67)	42(67)	0.000	0.000	0.982
**Hepatic encephalopathy No. (%)**	–	0(0)	18(4)	12(19)	0.007	0.000	0.000
**Upper gastrointestinal bleeding No. (%)**	–	0(0)	6(1)	5(8)	0.093	0.005	0.000
**28-day transplant-free mortality No. (%)**	–	0(0)	13(3)	11(18)	0.010	0.000	0.000
**3-month transplant-free mortality No. (%)**	–	0(0)	22(5)	19(30)	0.000	0.000	0.000

Data are expressed as mean ± standard deviation (SD), median (interquartile range) or number (percent). ACLF, acute-on-chronic liver failure; HBV, hepatitis B virus; ALT, alanine aminotransferase; AST, aspartate Aminotransferase. Comparisons between cohorts were performed by the Mann-Whitney u test or a Chi-square test.

We excluded patients over 75 years of age and patients with confirmed or suspected malignancy, pregnancy, previous organ transplantation, severe extra-hepatic diseases, or active bacterial infections.

The study complied with the principles of the Declaration of Helsinki and was approved by the Ethics Committee of Zhejiang University. Written consent was obtained from each patient or his/her authorized representative(s).

### Isolation of Circulating Neutrophils

Peripheral whole blood was drawn *via* venepuncture. Neutrophils were isolated using Polymorphprep (Axis-Shield PoC AS, Norway) by density centrifugation according to the manufacturer’s instructions. Then neutrophils were stained using CD11b-PE (Biolegend, USA) antibodies and then analyzed by flow cytometry (Canto-II, BD, USA). The purity of neutrophils exceeded 95% (**Supplementary Figure 1**). The viability of the neutrophils was examined by Countess™ II Automated Cell Counter (ThermoFisher Scientific, MA, USA) and Trypan Blue Stain (0.4%) (ThermoFisher Scientific, MA, USA) and found to be more than 90%.

### Analysis of Surface Markers

After the erythrocytes were lysed, whole blood samples were stained using TLR1-PE, TLR2-AF647, TLR4-PE, CXCR1-PE, CXCR2-FITC, FPR1-APC, CD62L-PE, CD43-FITC, CD11b-PE, CD64-APC (Biolegend, USA), CEACAM-1-PE (R&D, USA), and CD16-BV510 (Sony, USA) antibodies and then analyzed by flow cytometry (Canto-II, BD, USA). Isotype-matched immunoglobulins were used as negative controls.

### Transcriptomic Sequencing and Bioinformatic Analysis

Total RNA was extracted from freshly isolated neutrophils using TRIzol reagent (Invitrogen, CA, USA). The sequencing library was established using an NEBNext^®^ Ultra™ RNA Library Prep kit for Illumina^®^ (NEB, USA) and then sequenced on the Illumina HiSeq Xten platform according to the manufacturer’s instructions.

Clean data were obtained by removing low-quality reads or reads containing adaptor/poly-N sequences from the raw data. Differentially expressed genes (DEGs) between groups were identified using the DESeq R package (1.10.1), and genes with an adjusted P value less than 0.05 were considered differentially expressed. Gene pathway enrichment analysis of the DEGs was performed using the Reactome database; a pathway with a corrected P value less than 0.05 was considered significantly enriched in DEGs. All manipulations and statistical analyses were implemented using R freeware.

### Oxidative Burst Assay

The quantification of the oxidative burst activity of neutrophils was assessed using a Phagoburst kit (Glycotope Biotechnology GmbH, Heidelberg, Germany). Briefly, 100 µl of heparinized human whole blood activated or not activated for 15 min at 37°C with *E. coli*. After lysis of erythrocytes, fixation and washing, cells were collected for flow cytometry. Neutrophils were gated using forward scatter (FSC) and side scatter (SSC). The percentage of cells producing reactive oxygen species (ROS) and their mean fluorescence intensity (MFI) was calculated.

### Phagocytosis Assay

The phagocytosis capacity of neutrophils was assessed using a Phagotest kit (Glycotope Biotechnology GmbH, Heidelberg, Germany) according to the manufacturer’s instructions. In brief, 100μl of the anticoagulated whole blood sample was incubated with FITC-labeled *E. coli* bacteria for 15 min at 37°C. The reaction was quenched using brilliant blue. Cells were washed, resuspended in phosphate-buffered saline (PBS) and collected for flow cytometry. Neutrophils were gated and analyzed as described above. To determine the effect of plasma on neutrophil phagocytosis, healthy human neutrophils (1*10^6^) were isolated and cultured separately with plasma from healthy volunteers (100 μl) or ACLF patients (100 μl), and then the neutrophil phagocytosis was tested by the method above.

### Intracellular Myeloperoxidase (MPO) Assay

One hundred microliters of the anticoagulated blood sample were incubated with *E. coli* (3*10^8^/ml) for 30 min at 37°C. The cells were fixed and permeabilized afterwards. Then, the cells were stained with anti-MPO-FITC (MPO Flow Kit, Biolegend, USA) for 30 min at room temperature and washed and analyzed by flow cytometry.

### NET Assay

Whole blood samples (100 μl) were stimulated with opsonized *E. Coli* (3*10^8^/ml), fMLP (0.83 µmol/L) or PMA (1.35 µmol/L). An anti-human FITC-MPO antibody (20 µl/test) and SYTOX Red (10 nmol/L) (Biolegend, USA) were added and the cells incubated for 30 min at room temperature. After lysis of the erythrocytes, the cells were analyzed using flow cytometry (Canto-II, BD, USA). Neutrophils were selected by FSC and SSC, and NET formation was defined as MPO and SYTOX Red double-positive, as previously described (19). Healthy human neutrophils were isolated (1*10^6^), and co-cultured separately with plasma from healthy volunteers (100 μl) or ACLF patients (100 μl) for 3 h for determining the effect of plasma on neutrophil NET formation. Hereafter, the samples were stimulated with opsonized E. Coli (3*10^8^/ml) for 1 h and were tested the neutrophil NET production by the above method.

### Imaging of NET Formation

One hundred microliters of heparinized blood in the presence or absence of *E. coli* (3*10^8^/ml) were incubated for 1 h at 37°C. Then, an anti-human FITC-MPO antibody (20 µl/test) and SYTOX Red (10 nmol/L; Biolegend, USA) were added and incubated for 30 min at room temperature. Images were acquired on an ImageStream Multispectral Imaging Flow Cytometer (Amnis, part of EMD Millipore, USA) using the 40× magnification objective, which provides a numerical aperture (NA) of 0.75 and a pixel dimension of 0.5 m × 0.5 m.

A core diameter of 7 μm was used to maximize in-focus events. The 488 nm and 642 nm excitation laser were used at an output power of 150 mW. Objects with a minimum cross-sectional area of 50 m^2^ and a maximum of 600 m^2^ were collected to avoid acquiring debris or cellular aggregates. Typical files contained images of 20,000 cells. Cell images were analysed using IDEAS software, version 6.2. Cells in best focus were selected using the feature Brightfield (BF) Gradient RMS, a measurement of image contrast that excludes out-of-focus events. Doublets, aggregates, dead cells, and debris were excluded using SSC intensity and Syto intensity, and all analyses were restricted to single cells.

### Cytokine Assay

Purified neutrophils (1 × 10^6^/ml) were stimulated with 100 ng/ml LPS (Sigma-Aldrich, Steinheim, Germany) for 4 h at 37°C. In the collected supernatants, the levels of IL-2, IL-4, IL-6, IL-4, IL-10, TNF-α and IFN-γ were measured using a Cytometric Bead Array (CBA) (eBioscience, USA) following the manufacturer’s instructions. The lower limits of detection of the test for the cytokines were 2.6 pg/ml for IL-2 and IL-4, 3.0 pg/ml for IL-6, 2.8 pg/ml for IL-10 and TNF-α, and 7.1 pg/ml for IFN-γ.

The concentrations of plasma GM-CSF, MIG, IL-8, MCP-1, IP-10, G-CSF, IL-6 and IL-10 were also determined by CBA. The lower limits of the test for the detection of the cytokines were 0.2 pg/ml for GM-CSF, 1.1 pg/ml for MIG, 1.2 pg/ml for IL-8, 1.3 pg/ml for MCP-1, 0.5 pg/ml for IP-10, 1.6 pg/ml for G-CSF, 1.6 pg/ml for IL-6 and 0.13 pg/ml for IL-10.

### Glucose Uptake Assay

Neutrophils (1*10^6^) cultured in serum-free RPMI were supplemented with the fluorescent glucose analogue 2-(N-(7-nitrobenzen-2-oxa-1, 3- diazol-4-yl) amino)-2-deoxyglucose (2-NBDG) (Thermo Fisher Scientific, Inc.), at a final concentration of 500 µM. Then the neutrophils were left untreated or activated with opsonized E. Coli (3*10^8^/ml), and then incubated at 37°C for 4 h; next, they were washed with PBS and assessed by flow cytometry (Canto-II, BD, USA).

### Glut1 Expression

Whole blood samples (100 μl) were stimulated with opsonized E. Coli (3*10^8^/ml) or left untreated for 2 h. Then, samples were incubated with Mouse Anti-Human Glut1 PE conjugated Monoclonal Antibody (R&D) for 30 min at 4°C. After washing, cells were resuspended in PBS and assessed for Glut1 expression by flow cytometry (FACS Aria III; BD Biosciences). Isotype-matched immunoglobulins were used as negative controls.

### Pharmacological Inhibition of Glycolysis

Neutrophils (1*10^6^) were incubated in RPMI and then supplemented with 2-deoxyglucose (2-DOG) (Abmole, USA) at concentration of 2 mm to inhibit glycolysis for 15 min in a 5% CO2 atmosphere. Then, opsonized E. Coli (3*10^8^/ml) was added for another 3-h stimulation. After stimulation, cells were collected and tested for NET production as mentioned above.

### Endotoxin Measurement

Blood samples were collected in pyrogen-free heparinized tubes that were dry-baked at 120°C for 48 h. The plasma endotoxin level was determined by a Kinetic Turbidimetric LAL Kit for Endotoxin Detection (Xiamen Bioendo Technology Co., Ltd, China) as previously described (20).

### Statistical Analysis

All data were analysed using SPSS 20.0 for Windows (SPSS, Chicago, IL, USA), and a two-sided P value < 0.05 was considered statistically significant. Continuous data were compared using One-way ANOVA (LSD method for *Post-hoc* analysis), Student’s t-test or the Mann-Whitney u-test. Nominal variables were compared using a chi-square test. A Cox proportional hazard model was used to explore risk factors of adverse outcomes.

## Results

### Neutrophil Quantification

**Figure 1:**
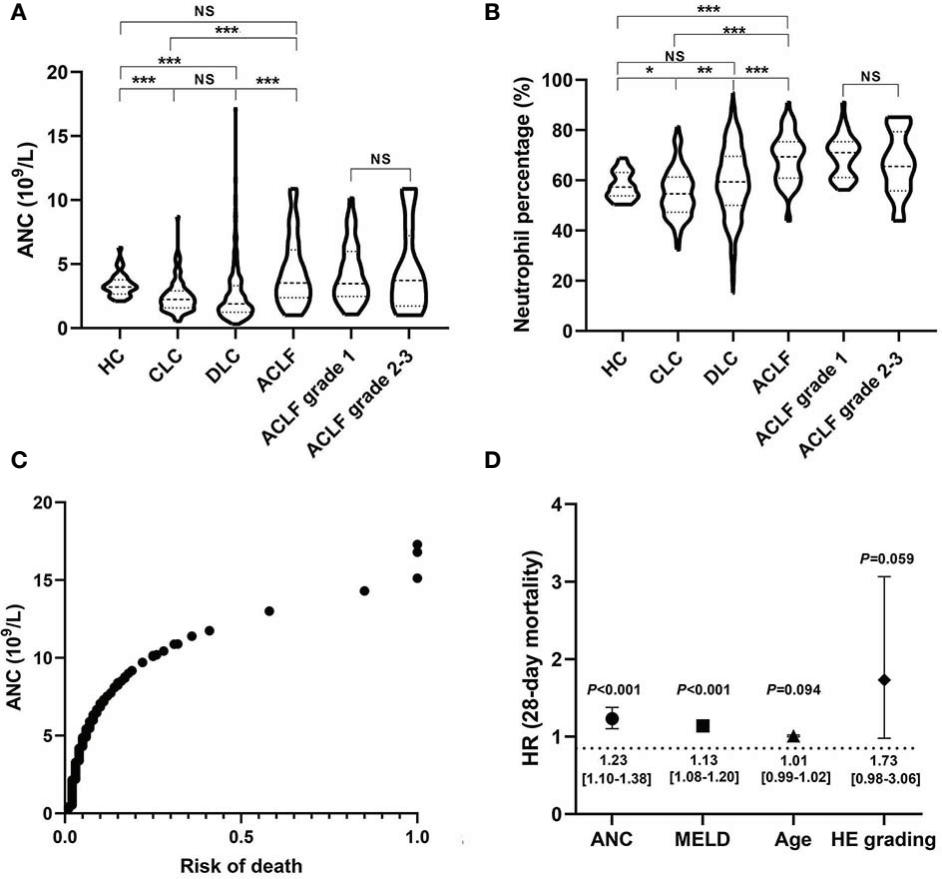
Circulating neutrophil quantity and viability alterations in hepatitis B virus related acute-on-chronic liver failure (HBV-ACLF) patients. **(A, B)** Comparison of absolute counts and percentages of circulating neutrophils among patients with HBV-ACLF (N=63), decompensated liver cirrhosis (DLC) (N=475), compensated liver cirrhosis (CLC) (N=95), and healthy controls (HCs) (N=34), as well as the comparison between early ACLF (grade 1; N=44) and advanced ACLF (grade 2-3; N=19). Statistical analysis was performed by One-way ANOVA (LSD method for *Post-hoc* analysis) or Student t test. Data are expressed as mean ± standard deviation (SD). **(C)** The association between absolute neutrophil count (ANC) and 28-day risk of death in patients with HBV-related cirrhosis (N=633). Statistical analysis was performed by Cox proportional hazard model. **(D)** Independent 28-day risk factors for death in patients with HBV-related cirrhosis and their risk estimates with 95%CI. Statistical analysis was performed by the Mann-Whitney u test. Data are expressed as mean ± standard deviation (SD). HE, Hepatic encephalopathy; MELD, Model end-stage liver disease. NS, P > 0.05; *0.01 ≤ P < 0.05; **0.001 ≤ P < 0.01; ***P < 0.001.

In a large retrospective cohort of patients with HBV-associated cirrhosis, we demonstrated that both the absolute number of circulating neutrophils (4.33 ± 2.64*10^9/L) and the percentage (68.66 ± 9.85%) in patients with ACLF were significantly higher than that in patients with CLC (2.48 ± 1.29*10^9/L, 54.94 ± 9.68%, both P<0.001) and DLC (2.62 ± 2.27*10^9/L, 59.26 ± 14.03%, both P<0.001) (**Figures 1A, B**). However, no significant differences were found between patients with early (grade 1) and advanced (grade 2-3) ACLF (**Figures 1A, B**). Subsequently, we found that the ANC was positively correlated with the risk of death (**Figure 1C**) as well as a strong risk factor associated with 28-day short-term mortality in patients with cirrhosis [HR (95%CI):1.23 (1.10–1.38), P<0.001)], even when adjusted by age, HE and MELD score (**Figure 1D**). Additionally, there was a significant correlation between baseline neutrophil count and development of bacterial infections in patients with cirrhosis (OR=1.19, 95%CI 1.06–1.34, P=0.002).

### Neutrophil Phenotypes

The phenotypes of circulating neutrophils were compared, and a variety of surface receptors, including TLRs, chemokine receptors, adhesion molecules, and activation markers, were investigated by flow cytometry. As shown in (**Table 2**), we found that the expression of TLR-1, TLR-4, and CEACAM-1 on neutrophils from ACLF patients was significantly increased, but the expression of TLR-2 and FPR1 on ACLF-patient neutrophils was significantly decreased compared to that on CLC patient or HC neutrophils.

**Table 2:** Neutrophil phenotypes in Cohort B.

Surface markers	HC (N=11)		C-LC (N=14)		ACLF (N=16)	
	Percentage	MFI	Percentage	MFI	Percentage	MFI
TLRs						
**TLR-1**	62.7 ± 20.8%	415.9 ± 177.2	51.2 ± 16.5%	365.1 ± 105.5	68.1 ± 13.7%**^#^**	434.1 ± 148.8
**TLR-2**	77.0 ± 14.9%	785.3 ± 256.6	79.8 ± 11.9%	795.5 ± 233.1	68.1 ± 11.2%*^#^	638.9 ± 170.6**^#^**
**TLR-4**	29.5 ± 12.8%	275.6 ± 69.2	24.9 ± 12.6%	255.6 ± 72.9	35.2 ± 11.6%**^#^**	296.1 ± 63.2
Chemokine Receptors						
**CXCR1**	99.7 ± 0.3%	8,732.3 ± 3,337.8	98.9 ± 1.6%	7,290.1 ± 2,394.1	99.2 ± 0.8%	7,442.7 ± 2,661.6
**CXCR2**	99.6 ± 0.3%	1,409.6 ± 560.5	98.7 ± 1.1%	1245.4 ± 300.8	99.4 ± 0.4%	1,204.3 ± 270.9
**FPR1**	82.3 ± 14.1%	1,3634 ± 5,241	91.9 ± 9.4%^※^	13,803 ± 4,618	73.5 ± 24.2%**^#^**	1,0215 ± 6,030
Adhesion receptors						
**CD62L**	67.5 ± 20.0%	1,114.1 ± 525.1	72.3 ± 19.7%	2,496.1 ± 4,472.7	71.1 ± 22.9%	2,010.1 ± 1,757.8
**CD43**	81.6 ± 21.9%	3,330.3 ± 1,020.8	78.9 ± 23.7%	2,847.6 ± 791.5	82.1 ± 12.8%	2,765.7 ± 700.9
**CD11b**	99.9 ± 0.06%	13,322 ± 7,368	99.8 ± 0.2%	1,1252 ± 4,523	99.7 ± 0.8%	12,555 ± 4,381
**CEACAM-1**	40.4 ± 27.6%	528.4 ± 282.4	29.6 ± 15.1%	403.7 ± 138.3	54.0 ± 23.4%**^#^**	673.7 ± 320.3**^#^**
Activation markers						
**CD16**	97.3 ± 3.5%	31,897 ± 31,245	96.2 ± 4.8%	34,960 ± 17,191	97.2 ± 3.3%	27,465 ± 12,619
**CD64**	62.4 ± 18.0%	986.2 ± 556.0	77.1 ± 11.6%	1,255.5 ± 685.2	72.5 ± 19.5%	1,158.6 ± 695.4

^#^represents a significant difference (P<0.05) between patients with ACLF and CLC; *represents a significant difference (P<0.05) between patients with ACLF and HC. ^※^represents a significant difference (P<0.05) between patients with CLC and HC. Comparisons between groups were performed by One-way ANOVA (LSD method for Post-hoc analysis). Data are expressed as mean ± standard deviation (SD).

### Neutrophil Transcriptional Reprogramming

**Figure 2:**
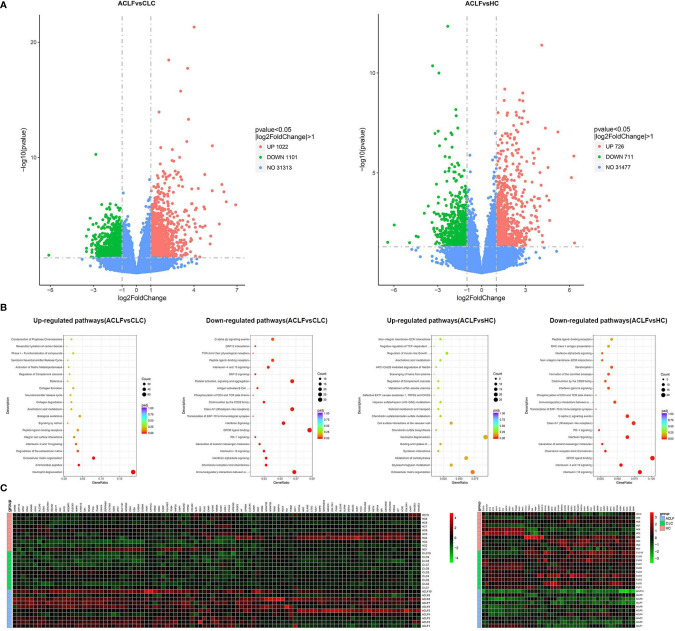
Neutrophil transcriptomics in hepatitis B virus related acute-on-chronic liver failure (HBV-ACLF) patients. **(A)** The volcano graph showed differentially expressed genes of neutrophils between patients with HBV-ACLF (N=10) and compensated liver cirrhosis (CLC) (N=10) or healthy controls (HCs) (N=10). Red indicates up-regulation of expression, and green indicates down-regulation of expression. **(B)** Reactome analysis showed that down-regulated and up-regulated pathways between patients with HBV-ACLF (N=10) and CLC (N=10) or HCs (N=10). **(C)** Heatmap representations of the most down-regulated and up-regulated genes in ACLF-neutrophils compared to CLC-neutrophils; Red indicates up-regulation of expression, and green indicates down-regulation of expression.

The transcriptomic profiles of circulating neutrophils were compared between patients with ACLF (N=10), patients with CLC (N=10) and HC (N=10). Compared with CLC-neutrophils, the expression of 1022 genes were found to be upregulated in ACLF-neutrophils, and 1101 genes were downregulated (**Figure 2A**). And compared to HC, the expression of 726 genes were up-regulated in ACLF-neutrophils, and 711 genes were down-regulated (**Figure 2A**).

The pathway analysis identified multiple pathways enriched or down-regulated in ACLF-neutrophils when comparing with neutrophils from CLC or HC groups. For example, the pathway analysis identified multiple pathways enriched or down-regulated in ACLF-neutrophils when comparing with neutrophils from CLC or HC groups, e.g., genes associated with degranulation (representative genes such as myeloperoxidase, cathepsin G and elastase), antibacterial immunity (representative genes such as defensin alpha 1 and defensin alpha 4), and extracellular matrix remodeling (representative genes such as matrix metallopeptidase 8 and matrix metallopeptidase 9) were significantly enriched in neutrophils from ACLF patients in comparison with CLC-neutrophils (**Figures 2B, C**). In contrast, the expression of genes associated with interactions with adaptive immunity (representative genes such as killer cell lectin-like receptor G1 and cytotoxic and regulatory T cell molecule), chemokine production (representative genes such as C-X3-C motif chemokine receptor 1, C-X-C motif chemokine ligand 8 and C-C motif chemokine receptor 5) and the IFNα/β pathway (representative genes such as interferon-induced protein with tetratricopeptide repeats 3, interferon-induced protein with tetratricopeptide repeats 1 and interferon alpha-inducible protein 6) was significantly downregulated (**Figures 2B, C**).

### Neutrophil Functional Aberrance

**Figure 3:**
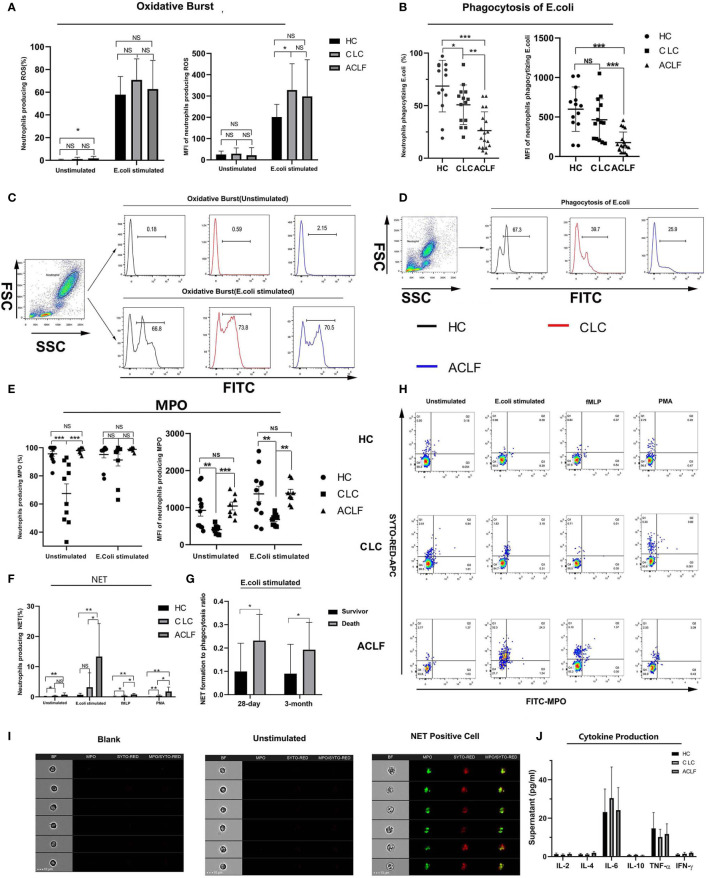
Multiple neutrophils functions in hepatitis B virus related acute-on-chronic liver failure (HBV-ACLF) patients. **(A, C)** The percentage and mean fluorescence intensity (MFI) of neutrophils producing reactive oxygen metabolites with or without E.coli stimulation in patients with HBV-ACLF (N=18), compensated liver cirrhosis (CLC) (N=14) or healthy control (HC) (N=13) and the representative graph showing the gating strategy, Data are expressed as mean ± standard deviation (SD). **(B, D)** The percentage and MFI of neutrophils phagocytizing E.coli in patients with HBV-ACLF (N=18), CLC (N=14), and HC (N=13) and the representative graph showing the gating strategy, Data are expressed as mean ± standard deviation (SD). **(E)** The percentage and MFI of neutrophils producing intracellular myeloperoxidase (MPO) in patients with HBV-ACLF (N=8), CLC (N=10), or HC (N=11). Data are expressed as mean ± standard error of mean (SEM). **(F, H)** The percentage of neutrophils producing NET in patients with HBV-ACLF (N=7), CLC (N=6), and HC (N=8) and the representative graph showing the gating strategy, Data are expressed as mean ± standard deviation (SD). **(G)** The ratio of percent of neutrophil extracellular trap (NET) producing neutrophils to that of phagocytic neutrophils in deceased ACLF patients or survivors at 28-day (deaths: four cases, survivors: 18 cases) or 3-month (deaths: six cases, survivors: 15cases). Data are expressed as mean ± standard deviation (SD). **(I)** Imaging flow of NET formation. The four columns showed the morphology, MPO fluorescence, SYTOX RED fluorescence and MPO-SYTOX RED merged fluorescence of single selected neutrophil. **(J)** Supernatant levels of IL-2, IL-4, IL-6, IL-10, TNF-α, and IFN-γ produced by neutrophils from patients with HBV-ACLF (N=7), CLC (N=5), and HC (N=6) after stimulated by lipopolysaccharide (LPS). Data are expressed as mean ± standard error of mean (SEM). Statistical analysis was performed by the Mann-Whitney u test. NS, P > 0.05; *0.01 ≤ P < 0.05; **0.001 ≤ P < 0.01; ***P < 0.001.

Next, we tested whether multiple neutrophil functions, including oxidative burst, phagocytosis, degranulation, NET formation and cytokine production, were altered in ACLF patients. After stimulation with E. coli, a marked stepwise decrease in phagocytosis was observed across the three groups (percentage: HC: 68.61 ± 24.55%, CLC: 50.79 ± 18.81%, ACLF: 26.22 ± 17.93%; MFI: HC: 599.38 ± 281.64, CLC: 466.21 ± 264.54, ACLF: 175.33 ± 134.38: **Figures 3B, D**). In contrast, the stepwise increase in NET formation in response to either E. coli, fMLP or PMA showed a sharp increase in ACLF patients (percentage after E. coli stimulation: 13.34 ± 10.92%; **Figures 3F, H**). The morphological features of NET-forming neutrophils are shown in **Figure 3I**.

No significant differences in oxidative burst capacity were found between the ACLF and CLC or HC groups, except for a moderate increase in spontaneous oxidative burst in ACLF patients compared to HC (P =0.019; **Figures 3A, C**). Then we found a significantly increased intracellular MPO level, as indicated by either percentage (ACLF vs CLC, P=0) or MFI (ACLF vs CLC, P=0), in ACLF patients compared to patients with CLC but not compared to HC (**Figure 3E**), which suggests changes of neutrophil degranulation function during disease development, but further experiments and exploration are still needed to determine this. In addition, the ratio of percent of NET formation to phagocytosis was significantly higher in deceased ACLF patients than survivors (deceased at 28-day: 1.00 ± 0.03, survivors at 28 -day: 0.23 ± 0.06, P=0.027, Survival at 3-month: 0.09 ± 0.03, Death at 3-month: 0.19 ± 0.05, P=0.024: **Figure 3G**). After LPS stimulation, neutrophils produced detectable but low levels of cytokines such as IL-2, IL-4, IL-6, IL-10, TNF-α, and IFN-γ, and no significant differences were found among the groups (**Figure 3J**).

### Circulation Environment

**Figure 4:**
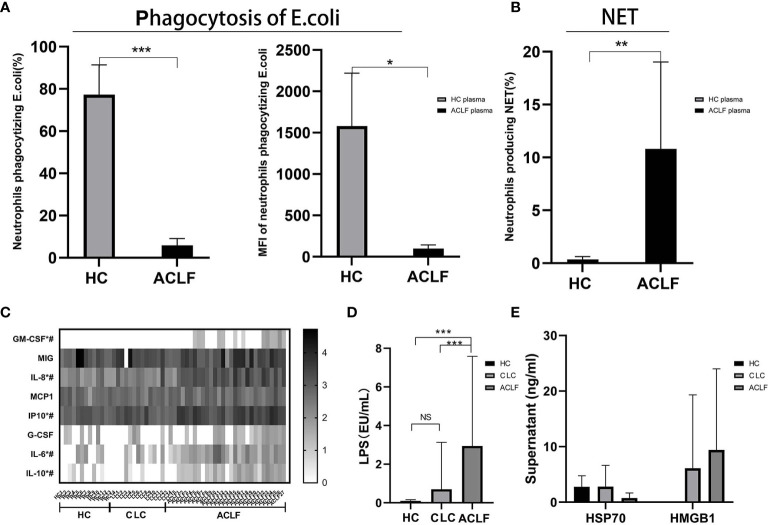
Circulation environment in hepatitis B virus related acute-on-chronic liver failure (HBV-ACLF) patients. **(A)** The percentage and mean fluorescence intensity (MFI) of neutrophils phagocytizing E.coli of healthy controls incubated with the plasma of ACLF(N=5) and healthy control **(HC)** (N=5). **(B)** The percentage of neutrophils producing neutrophil extracellular trap (NET) of healthy controls incubated with the plasma of ACLF (N=5) and HC (N=5). **(C)** Comparison of plasma levels of inflammatory mediators GM-CSF, MIG, IL-8, MCP-1, IP-10, G-CSF, IL-6, and IL-10 among patients with HBV-ACLF (N=27), CLC (N=15), or HC (N=14). * Represents a significant difference (P<0.05) between patients with ACLF and CLC; # represents a significant difference (P<0.05) between patients with ACLF and HC. **(D)** Comparison of plasma lipopolysaccharide (LPS) among patients with HBV-ACLF (N=22), CLC (N=18), or HC (N=16). **(E)** Comparison of DAMP molecules HMGB-1 and HSP among patients with HBV-ACLF (N=6), CLC (N=9), or HC (N=8). Data are expressed as mean ± standard deviation (SD). Statistical analysis was performed by the Mann-Whitney u test. NS, P > 0.05; *0.01 ≤ P < 0.05; **0.001 ≤ P < 0.01; ***P < 0.001.

We hypothesized the circulating neutrophil dysfunction in ACLF patients may result from their distinct circulatory environment. The plasma co-culture experiments revealed that healthy neutrophils became less phagocytic (percentage: HC plasma: 77.28 ± 14.08%, ACLF plasma: 5.9 ± 3.21%, P=0; MFI: HC plasma: 1581 ± 638.66, ACLF plasma: 99.8 ± 43.25, P=0.043: **Figure 4A**), but produced more NET (percentage: HC plasma: 0.36 ± 0.26%, ACLF plasma:10.8 ± 8.22%, P=0.009: **Figure 4B**) in stimulation to E. coli after incubation with ACLF plasma, a functional phenotype mimicking ACLF-neutrophils. Then, the components of circulatory environment underlying neutrophil dysfunction was investigated. Levels of inflammatory mediators, including GM-CSF, IL-6, IL-8, IL-10, and IP-10, were significantly increased in ACLF patients (**Figure 4C**). Additionally, the concentration of the bacterial product LPS was markedly increased in the circulation of patients with ACLF (**Figure 4D**). However, elevated concentrations of the DAMP molecules HMGB-1 and HSP were not seen in ACLF patients (**Figure 4E**).

### The Effect of Glycolysis Inhibition on NET Formation

**Figure 5:**
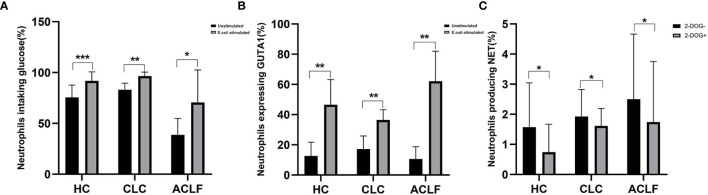
The effect of glycolysis inhibition on neutrophil extracellular trap (NET) formation. **(A)** The percentage of neutrophils uptaking glucose in patients with hepatitis B virus related acute-on-chronic liver failure (HBV-ACLF) (N=5), compensated liver cirrhosis (CLC) (N=6), and healthy controls (HCs) (N=8) following E. coli stimulation. **(B)** The percentage of neutrophils expressing Glut1 in patients with HBV-ACLF (N=5), CLC (N=7), and HCs (N=6) following E. coli stimulation. **(C)** After additional supplement of 2-DOG, the percentage of neutrophils producing NET was determined from patients with HBV-ACLF (N=9), CLC(N=7), and HCs (N=7). Data are expressed as mean ± standard deviation (SD). Statistical analysis was performed by the Mann-Whitney u test. NS, P > 0.05; *0.01 ≤ P < 0.05; **0.001 ≤ P < 0.01; ***P < 0.001.

A recent study has shown that glycolysis is required for NET formation in healthy neutrophils and therefore we tested whether inhibiting glycolysis is able to correct over-

production of NET in ACLF-patient neutrophils. After E. coli stimulation, glucose uptake and transport (as indicated by Glut1 expression) were markedly enhanced in neutrophils from either HC, CLC, or ACLF patients (**Figures 5A, B**). 2-DOG, an inhibitor of glycolysis, significantly reduced NET formation of neutrophils either in patients with ACLF (Percentage of NET-producing neutrophils: 1.81 ± 1.84% vs 1.11 ± 1.61%, P=0.012), CLC (1.93 ± 0.90% vs 1.61 ± 0.58%, P=0.042), or controls (1.57 ± 1.47% vs 0.74 ± 0.93%, P=0.028) (**Figure 5C**).

## Discussion

Neutrophils are key effector cells of host innate immunity and play a critical role in resisting bacterial infections and dealing with tissue damage (21). HBV-ACLF is an innate immune-mediated and systemic inflammation-driven syndrome characterized by multi-organ failure/dysfunction and is frequently complicated by bacterial infection (3, 22). The present study revealed diverse alterations in the quantity, phenotype, transcription, and function of neutrophils from ACLF patients. The findings of this study suggest that the immunological feature of ACLF is intensive immune system activation with concurrent immune paresis.

The most remarkable functional aberrance of ACLF-patient neutrophils was a significant decrease in phagocytic activity but an increase in NET formation in response to bacterial stimulation. Neutrophils kill microbes *via* phagocytosis, oxidant species generation, and cell proteolytic machinery activation (23). The formation of NETs represents an alternative mechanism to defend against invading pathogens. The formation and release of sticky web-like structures composed of decondensed DNA decorated with citrullinated histone and neutrophil granule proteins enable neutrophils to catch and kill pathogens (24). We hypothesize that the increase in NET formation is a compensatory mechanism for the impaired phagocytic capacity. When phagocytosis is weakened or ineffective, the release of NETs prevents microbes from escaping immunity (25).

The neutrophil transcriptomics demonstrated several major transcriptional reprogramming signatures of ACLF-patient neutrophils. The active transcription of degranulation-related genes concurs with the increased propensity of neutrophils to produce NETs, the formation of which requires neutrophil granule proteins, such as MPO and neutrophil elastase. Second, the enrichment of ECM-related genes suggests the role of neutrophils in tissue repair. In contrast, the expression of genes associated with interactions with adaptive immunity was significantly suppressed. As the formation of NET in neutrophils is elevated in ACLF patients but not those with CLC, we hypothesize neutrophil functional reprogramming may be shaped by the circulatory microenvironment in ACLF, as confirmed by the incubation experiment showing that healthy neutrophils acquired a functional phenotype like neutrophils from ACLF patients after co-culture with ACLF plasma. It is not clear, however, the specific components in ACLF plasma contribute to neutrophil dysfunction. The prevailing hypothesis is that systemic innate immune cells are activated by pathogen-associated molecular patterns (PAMPs) produced and released from the leaking intestinal tract, as well as by DAMPs released from necrotic hepatocytes through pattern recognition receptors, such as TLRs and nucleotide-binding oligomerization domain-like receptors (NLRs) (26). In our study, however, the levels of DAMP molecules, such as HMGB-1 and HSP70, were not significantly elevated. Nevertheless, the role of DAMPs in inducing neutrophil activation cannot be excluded. The release of DAMPs into the circulation may occur at disease onset and would not be significant at later stages. Some DAMPs are insoluble but exposed on the cell surface. For example, HSPs may translocate to the cell surface after atypical cell death (27). Other DAMP molecules should be investigated. In contrast, the level of LPS was found to be significantly increased in the systemic circulation of HBV-ACLF patients. Our prior study also showed an increase in circulating bacterial DNA in HBV-ACLF patients (28). Furthermore, previous studies have shown that the effect of ACLF plasma on neutrophil phagocytic activity and resting oxidative stress is significantly attenuated after antibiotic treatment (10). All these findings suggest that persistent stimulation by PAMPs plays an important role in inducing neutrophil functional alterations in ACLF. In addition, the spill-over of inflammatory mediators into the circulation of ACLF patients may contribute to neutrophil functional alterations. IL-6 and IL-8, the levels of which were significantly elevated in the plasma of patients with ACLF, are reported to prime resting neutrophils (29). Therefore, a combination of inflammatory and microbial signals may cause neutrophil functional reprogramming in HBV-ACLF patients. Likewise, microbial signals may have impact on the neutrophil phenotypes. The increased expression of TLR-1 and TLR-4 may be associated with the neutrophil response to recognize enrichment of specific bacterial products such as LPS or bacterial lipoproteins and LTAs in circulation of HBV-ACLF patients. And subsequent TLR activation is reported to regulate chemokine receptor expression (30).

Functional changes in neutrophils have potential clinical implications. Previous studies have shown that impaired neutrophil phagocytosis is closely associated with an increased risk of bacterial infection in patients with cirrhosis or alcoholic hepatitis (10). In this study, a severe decline in phagocytic activity was found in ACLF-patient neutrophils. The functional defects of neutrophils are consistent with the increased susceptibility of ACLF patients to bacterial infection and the increased short-term mortality (31). At the same time, we found that neutrophil count was positively correlated with the risk of short-term death. It is well known that Granulocyte-colony stimulating factor (G-CSF) can expand neutrophil populations while improve the survival of ACLF patients at the same time (32, 33). These conclusions seem to be mutually contradictory, but in view of a previous study proving that G-CSF can reverse neutrophil phagocytosis, the contradiction can be partly explained by the dual influences of G-CSF on ACLF patient survival ratio (34). In addition, excessive NET formation may cause collateral immune damage (35–37). In contrast, the use of Granulocyte-colony stimulating factor (G-CSF), was reported to improve the survival of ACLF patients and the clinical benefit may be partially attributed to the effect of reversing neutrophil phagocytosis by G-CSF (32–34). Excessive NET formation may clog the pancreatic duct and induce aseptic inflammation in acute pancreatitis (38). NETs may promote thrombosis, and thus leading to microcirculatory disorders (39). Furthermore, NET components can stimulate monocytes/macrophages and amplify the inflammatory response (40). Therefore, skewed neutrophil function may be a hallmark feature of ACLF-associated immune dysfunction phenotypes, along with concurrent SI and immunosuppression, which predisposes ACLF patients to inflammation-related organ damage and an increased risk of bacterial infection. Our preliminary data has shown that the severity of neutrophil dysfunction, as indicated by the ratio of NET-producing cells to phagocytic cells upon E. coli challenge, correlated with the short-term outcome of ACLF patients. Nevertheless, further large, prospective studies are needed to validate the association.

It has been shown that innate immune cell function can be controlled at the metabolic level. A recent study has shown that glucose metabolism is actively involved in NET formation in healthy neutrophils (41). Concurrent with this study, we demonstrate here that glucose uptake and transport were activated both in health, CLC and ACLF neutrophils following stimulation, Further, blocking glycolysis using an inhibitor can reduce the capacity to produce NET in neutrophil derived from all the three groups. This finding suggested a markedly increase of glucose requirement for NET formation and the potential of metabolic rewiring of neutrophils in modifying the immunological function in ACLF patients.

In conclusion, circulating neutrophils from HBV-ACLF patients present quantitative, phenotypic, transcriptional, and functional alterations. The functional alterations are shaped by the specific ACLF circulatory microenvironment and associated with poor outcome. Further large studies should be performed to investigate the association between neutrophil dysfunction and disease progression, complications, and mortality. Additionally, the efficacy and safety of therapies targeting at glycolysis for reversing neutrophil dysfunction in ACLF merits further investigation.

## Data Availability Statement

The original contributions presented in the study are included in the article/**Supplementary Material**. Further inquiries can be directed to the corresponding authors.

## Ethics Statement

The study received approval from the Ethics committee of The First Affiliated Hospital of Zhejiang University (2017-421). The patients/participants provided their written informed consent to participate in this study.

## Author Contributions

YS, ZC, and JS conceived the study. WW and SS conceived and designed the experiments while WW, SS, YW, RZ, and HR performed them. YW, ZL, HZ, and YZ were involved in clinical data collection. YS, WW, SS, and YW wrote the manuscript. All authors contributed to the article and approved the submitted version.

## Funding

This research was funded by the National Natural Science Foundation of China (grant nos. 81671949, 81870425, and 81670567), the 13-5 State S&T Projects of China (2018ZX10302-206) and the Fundamental Research Funds for the Central Universities.

## Conflict of Interest

The authors declare that the research was conducted in the absence of any commercial or financial relationships that could be construed as a potential conflict of interest.
