# Supplementary material for: Circulating Neutrophil Dysfunction in HBV-Related Acute-on-Chronic Liver Failure
Source: Front Immunol. 2021 Feb 25;12:620365. doi: 10.3389/fimmu.2021.620365 (PMC7947208; doi:10.3389/fimmu.2021.620365)
Supplement: Supplementary file 1 [file DataSheet_1.pdf]

Supplementary Material

Supplementary Table 1 Baseline characteristics of study population in Cohort B

Variables	HC (N=21)	CLC (N=28)	ACLF (N=67)	P value (HCvsACLF)	P value (CLCvsACLF)
**Age (years)**	46.0(27.5)	52(10.8)	48(23)	0.509	0.083
**Male No. (%)**	9 (43)	24(86)	57(85)	0.000	0.936
**HBV DNA [Lg_{10} (copies/mL)]**	-	5(5.3)	4.9(2.7)	0.000	0.001
**HBeAg positivity No. (%)**	-	13(46)	28(42)	0.000	0.722
**ALT (IU/L)**	15(7)	32(25)	160(323)	0.000	0.000
**AST(IU/L)**	16(7)	34(30)	118(191)	0.000	0.000
**Albumin (g/L)**	42.2±1	39.7±1.0	31.6±0.5	0.000	0.000
**Serum bilirubin (μmol/L)**	7.0(5.0)	16.4(13.0)	326.9(184.5)	0.000	0.000
**INR**	1.0(0.1)	1.1(0.2)	1.9(0.9)	0.000	0.000
**Creatinine (μmol/L)**	67(8)	71(15)	63(20)	0.635	0.154
**Serum sodium (mmol/L)**	141(1)	142(4)	138(4)	0.000	0.000
**Platelet count (10^9/L)**	212(64)	97(87)	99(51)	0.000	0.784
**Ascites No. (%)**	-	0(0)	37(55)	0.000	0.000
**Hepatic encephalopathy No. (%)**	-	0(0)	11(17)	0.047	0.022
**28-day transplant-free mortality No. (%)**	-	0(0)	13(19)	0.005	0.001
**3-month transplant-free mortality No. (%)**	-	0(0)	15(23)	0.003	0.001

Data are expressed as mean ± standard deviation (SD), median (interquartile range) or number (percent). ACLF: acute-on-chronic liver failure; HBV: hepatitis B virus; ALT: alanine aminotransferase; AST: aspartate Aminotransferase; Comparisons between cohorts were performed by the Mann-Whitney u test or a Chi-square test.

Supplementary Figure 1 The Purity of Circulating Neutrophils

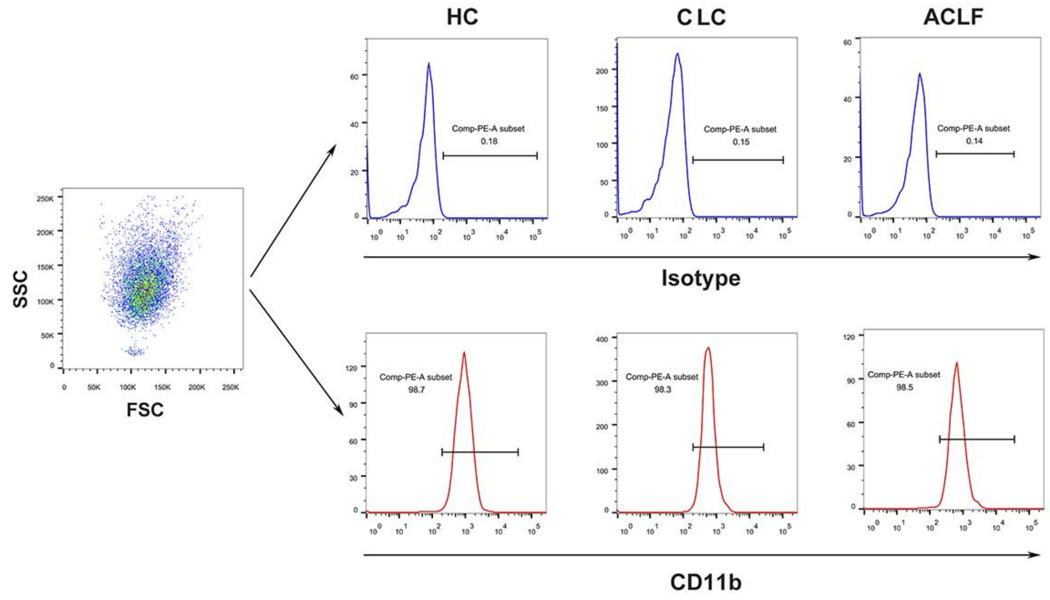

Supplementary Figure 1. The purity of circulating neutrophils. The representative graph showing the gating strategy in patients with HBV-ACLF, CLC or HC.
